# Machine learning model for the prediction of prostate cancer in patients with low prostate-specific antigen levels: A multicenter retrospective analysis

**DOI:** 10.3389/fonc.2022.985940

**Published:** 2022-08-18

**Authors:** Xiaobin Deng, Tianyu Li, Linjian Mo, Fubo Wang, Jin Ji, Xing He, Bashir Hussein Mohamud, Swadhin Pradhan, Jiwen Cheng

**Affiliations:** ^1^ Department of Urology, The First Affiliated Hospital of Guangxi Medical University, Nanning, China; ^2^ Institute of Urology and Nephrology, The First Affiliated Hospital of Guangxi Medical University, Nanning, China; ^3^ Center for Genomic and Personalized Medicine, Guangxi Medical University, Nanning, China; ^4^ Department of Urology, Changhai Hospital, Naval Medical University, Shanghai, China

**Keywords:** prostate cancer, prostate-specific antigen, diagnosis, machine learning, predictive model, real-world study

## Abstract

**Objective:**

The aim of this study was to develop a predictive model to improve the accuracy of prostate cancer (PCa) detection in patients with prostate specific antigen (PSA) levels ≤20 ng/mL at the initial puncture biopsy.

**Methods:**

A total of 146 patients (46 with Pca, 31.5%) with PSA ≤20 ng/mL who had undergone transrectal ultrasound-guided 12+X prostate puncture biopsy with clear pathological results at the First Affiliated Hospital of Guangxi Medical University (November 2015 to December 2021) were retrospectively evaluated. The validation group was 116 patients drawn from Changhai Hospital(52 with Pca, 44.8%). Age, body mass index (BMI), serum PSA, PSA-derived indices, several peripheral blood biomarkers, and ultrasound findings were considered as predictive factors and were analyzed by logistic regression. Significant predictors (P < 0.05) were included in five machine learning algorithm models. The performance of the models was evaluated by receiver operating characteristic curves. Decision curve analysis (DCA) was performed to estimate the clinical utility of the models. Ten-fold cross-validation was applied in the training process.

**Results:**

Prostate-specific antigen density, alanine transaminase-to-aspartate transaminase ratio, BMI, and urine red blood cell levels were identified as independent predictors for the differential diagnosis of PCa according to multivariate logistic regression analysis. The RandomForest model exhibited the best predictive performance and had the highest net benefit when compared with the other algorithms, with an area under the curve of 0.871. In addition, DCA had the highest net benefit across the whole range of cut-off points examined.

**Conclusion:**

The RandomForest-based model generated showed good prediction ability for the risk of PCa. Thus, this model could help urologists in the treatment decision-making process.

## Introduction

Prostate cancer (PCa) remains the most common malignancy in men. According to the latest cancer statistics published in 2022, PCa accounts for 27% of newly diagnosed malignancies in males, and is also the second leading cause of death among men with cancer (1). Serum total prostate-specific antigen (tPSA) is a specific tumor biomarker of PCa in the clinical setting. It has high tissue specificity, but is also associated with missed diagnoses and misdiagnoses (2, 3). A variety of benign diseases of the prostate, including benign prostatic hyperplasia and prostatitis, can lead to elevated serum tPSA levels (4, 5). In particular, tPSA levels in the range of 4 to 20 ng/mL are associated with a PCa incidence of less than 25%. In addition, patients with serum tPSA levels ≤4 ng/mL still carry the risk of PCa, and the detection rate in these patients may reach up to 20% (6, 7). Prostate puncture biopsy is currently the standard modality for diagnosing PCa, but as an invasive procedure, it carries a risk of infection. Moreover, the PCa detection rates on initial prostate puncture biopsies range from 23% to 42%. These limitations have greatly restricted its clinical use (8–11).

Given the limitations of the currently used diagnostic methods, a large number of studies are currently devoted to identifying new predictors of PCa. For example, PSA-derived indices, such as free-to-total PSA (F/T) values and prostate-specific antigen density (PSAD), have been found to exhibit greater diagnostic accuracy than PSA alone (12–14). In addition, several inflammatory and neurotrophic markers, including neutrophil-to-lymphocyte ratio, platelet-to-lymphocyte ratio, monocyte-to-lymphocyte ratio (15, 16), gamma-glutamyl transpeptidase-to-lymphocyte count ratio (17, 18), red cell distribution width-to-platelet ratio (19, 20), and alanine transaminase-to-aspartate transaminase ratio (21, 22), have previously been shown to have predictive value not only as inflammatory markers but also as indicators for the diagnosis and prognosis of malignancies. To date, studies focusing on the role of these composite indicators in the differential diagnosis of PCa are limited.

Machine learning (ML), as an important branch of artificial intelligence, can continuously optimize the performance of predictive or diagnostic models by learning and analyzing data, and can handle non-linear relationships better than traditional statistical scores. As a result, ML-based models have great potential for the diagnosis and prognosis of diseases (23–25). Therefore, our goal was to develop a new decision-support ML model based on real-world data for diagnosing PCa in patients with PSA levels ≤20 ng/mL.

## Materials and methods

### Ethics statement

This study was approved by the institutional review board of the First Affiliated Hospital of Guangxi Medical University. Written informed consent was obtained from all patients for the storage of their information for the purpose of research. All the research procedures were conducted in accordance with the Declaration of Helsinki.

### Data collection

Data from patients with PCa or benign prostatic hyperplasia who underwent systematic prostate puncture biopsy at our hospital between November 2015 and December 2021 were collected and retrospectively analyzed. We included adult patients with tPSA levels ≤20 ng/ml who underwent transrectal ultrasound (TRUS)-guided prostate puncture biopsy for at least systemic 12 cores with clear pathological results. The exclusion criteria were (1) a history of prostate cancer, prostate surgery, or 5-alpha-reductase inhibitor/drug for the treatment of endocrine dyscrasia in prostate cancer; (2) a diagnosis of prostatitis; (3) digital rectal examination (DRE), transrectal ultrasonography, or cystoscopy within two weeks before PSA detection (as these examinations may affect serum PSA levels); and (4) missing hematological data prior to puncture biopsy. Serum PSA concentrations (tPSA and fPSA) were measured before DRE and TRUS by enzyme-linked immunoassay. Prostate volume (PV) was calculated using the following formula:

PV = 0.52 × anterior/posterior diameter (cm) × left/right diameter (cm) × upper/lower diameter (cm)

TRUS was performed by experienced ultrasonologists.

### Statistical analysis

Continuous variables were converted into categorical variables. The optimum cutoff values obtained from ROC curve analysis were determined by maximizing the Youden index. Logistic regression analysis was applied to calculate the odds ratio (OR) with 95% confidence interval (CI). P < 0.05 was considered to indicate statistical significance. We used five different ML algorithms to analyze our data: logistic regression (LR), XGBoost (XGB), RandomForest (RF), multilayer perceptron (MLP), and k-nearest neighbor (kNN). After training, the model with the highest average AUC was chosen as the best algorithm. Furthermore, the ML-based model was tuned to avoid overfitting, and the accuracy of the algorithm was tested using the ten-fold cross-validation method. All variables were tested for Spearman correlations, and the results are presented as a heat map.

## Results

### Demographic features

**Figure 1:**
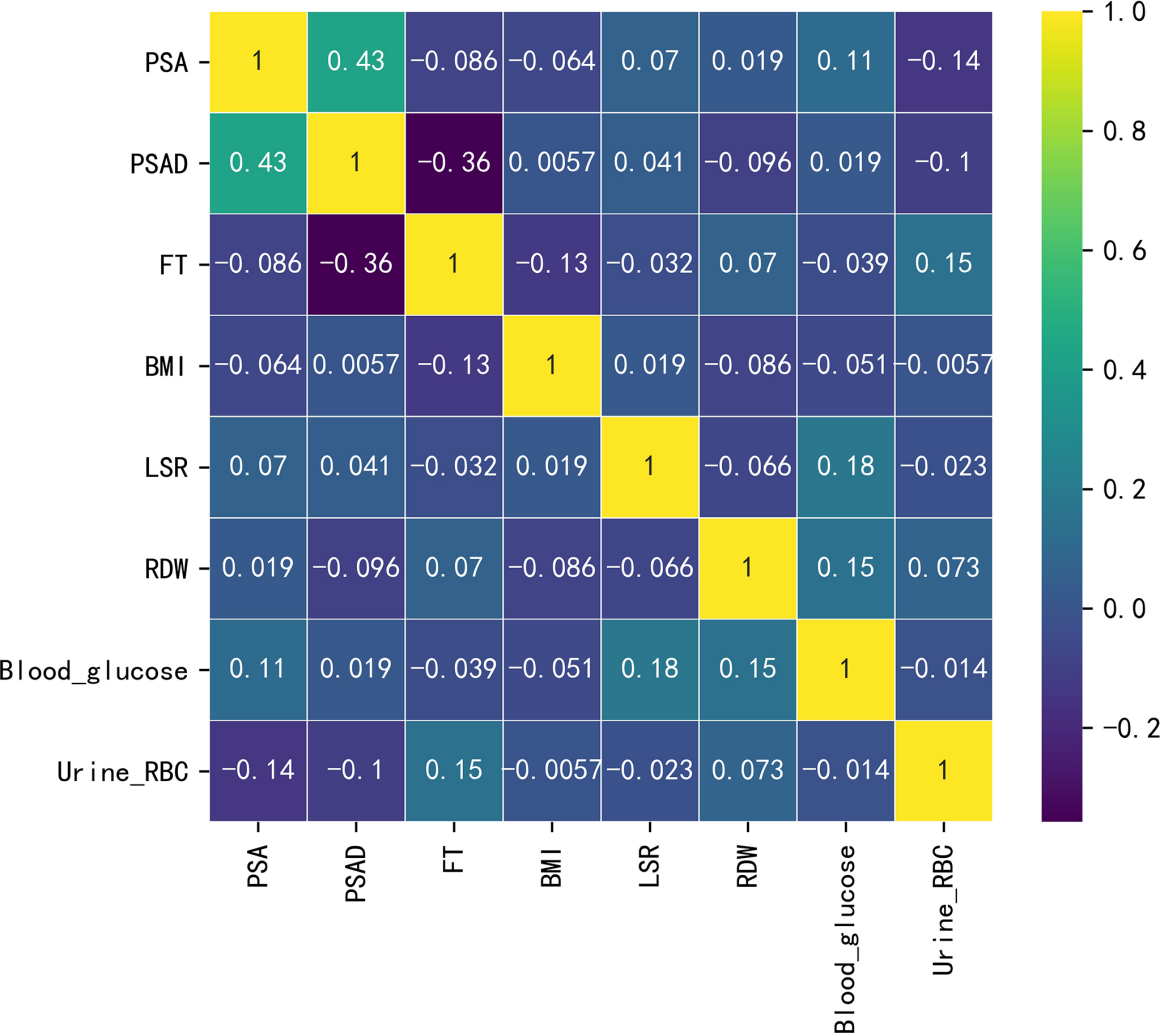
Heat map depicting the correlations between the examined variables.

A total of 146 eligible patients were included in this study. The optimal cut-off value of tPSA was 8.47 ng/mL, and the optimal cut-off value of BMI was 23.6 kg/m^2^. The detailed clinical characteristics of all the patients are presented in **Table 1**. Among the evaluated clinical characteristics, PSA, F/T, BMI, alanine transaminase-to-aspartate transaminase ratio (LSR), red cell volume distribution width (RDW), alkaline phosphatase (ALP), and urine RBC level were correlated with the risk of PCa. Based on the correlation heat map (**Figure 1**), eight highly correlated features were chosen as predictors. Weight is inextricably linked to BMI and, to a lesser degree, height. Therefore, we used BMI instead of height or weight, since it is a better indicator of obesity. The external validation cohort was screened based on inclusion and exclusion criteria consistent with the training cohort. Most of the externally validated variables did not differ statistically from the training cohort.

**Table 1:** Clinical characteristics of patients in the training cohort.

Variables, n (%)	Level	Total	BPH	PCa	P-value
*PSA;ng/mL*	<8.47	59 (40.411)	46 (46.000)	13 (28.261)	0.042
	≥8.47	87 (59.589)	54 (54.000)	33 (71.739)	
*fPSA;ng/mL*	<1.89	98 (67.123)	63 (63.000)	35 (76.087)	0.118
	≥1.89	48 (32.877)	37 (37.000)	11 (23.913)	
*F/T*	<0.103	31 (21.233)	15 (15.000)	16 (34.783)	0.007
	≥0.103	115 (78.767)	85 (85.000)	30 (65.217)	
*PV;mL*	<38.1	52 (35.616)	20 (20.000)	32 (69.565)	<0.001
	≥38.1	94 (64.384)	80 (80.000)	14 (30.435)	
*PSAD*	<0.24	95 (65.068)	80 (80.000)	15 (32.609)	<0.001
	≥0.24	51 (34.932)	20 (20.000)	31 (67.391)	
*Age;years*	<73	118 (80.822)	85 (85.000)	33 (71.739)	0.059
	≥73	28 (19.178)	15 (15.000)	13 (28.261)	
*BMI;kg/m²*	<23.62	75 (51.370)	57 (57.000)	18 (39.130)	0.045
	≥23.624	71 (48.630)	43 (43.000)	28 (60.870)	
*NLR*	<1.46	29 (19.863)	16 (16.000)	13 (28.261)	0.085
	≥1.46	117 (80.137)	84 (84.000)	33 (71.739)	
*PLR*	<131.01	87 (59.589)	62 (62.000)	25 (54.348)	0.381
	≥131.01	59 (40.411)	38 (38.000)	21 (45.652)	
*MLR*	<0.336	84 (57.534)	55 (55.000)	29 (63.043)	0.361
	≥0.336	62 (42.466)	45 (45.000)	17 (36.957)	
*GLR*	<13.21	60 (41.096)	37 (37.000)	23 (50.000)	0.138
	≥13.21	86 (58.904)	63 (63.000)	23 (50.000)	
*LSR*	<0.684	32 (21.918)	16 (16.000)	16 (34.783)	0.011
	≥0.684	114 (78.082)	84 (84.000)	30 (65.217)	
*RPR*	<0.00063	66 (45.205)	40 (40.000)	26 (56.522)	0.062
	≥0.00063	80 (54.795)	60 (60.000)	20 (43.478)	
WBC;*10⁹ */L*	<4.89	25 (17.123)	13 (13.000)	12 (26.087)	0.051
	≥4.89	121 (82.877)	87 (87.000)	34 (73.913)	
*Hb;g/dl*	<146.2	120 (82.192)	78 (78.000)	42 (91.304)	0.051
	≥146.2	26 (17.808)	22 (22.000)	4 (8.696)	
*RDW; %*	<0.15	125 (85.616)	81 (81.000)	44 (95.652)	0.019
	≥0.15	21 (14.384)	19 (19.000)	2 (4.348)	
Plt;*10⁹ */L*	<207	71 (48.630)	52 (52.000)	19 (41.304)	0.23
	≥207	75 (51.370)	48 (48.000)	27 (58.696)	
Neutrophil count;*10⁹ */L*	<4.14	84 (57.534)	54 (54.000)	30 (65.217)	0.203
	≥4.14	62 (42.466)	46 (46.000)	16 (34.783)	
Lymphocyte count;*10⁹ */L*	<1.5	38 (26.027)	23 (23.000)	15 (32.609)	0.219
	≥1.5	108 (73.973)	77 (77.000)	31 (67.391)	
Monocyte count;*10⁹ */L*	<0.44	32 (21.918)	19 (19.000)	13 (28.261)	0.209
	≥0.44	114 (78.082)	81 (81.000)	33 (71.739)	
Eosinophil count;*10⁹ */L*	<0.17	69 (47.260)	42 (42.000)	27 (58.696)	0.061
	≥0.17	77 (52.740)	58 (58.000)	19 (41.304)	
*Blood glucose;mmol/L*	<5	62 (42.466)	37 (37.000)	25 (54.348)	0.049
	≥5	84 (57.534)	63 (63.000)	21 (45.652)	
*γ-glutamyl transpeptidase;U*	<33.2	98 (67.123)	72 (72.000)	26 (56.522)	0.064
	≥33.2	48 (32.877)	28 (28.000)	20 (43.478)	
*Creatinine;μmol/L*	<88	86 (58.904)	62 (62.000)	24 (52.174)	0.262
	≥88	60 (41.096)	38 (38.000)	22 (47.826)	
*ALP;U*	<90	21 (67.742)	18 (78.261)	3 (37.500)	0.034
	≥90	10 (32.258)	5 (21.739)	5 (62.500)	
*Urine WBC*	Negative	116 (79.452)	75 (75.000)	41 (89.130)	0.05
	Positive	30 (20.548)	25 (25.000)	5 (10.870)	
*Urine RBC*	Negative	121 (82.877)	77 (77.000)	44 (95.652)	0.005
	Positive	25 (17.123)	23 (23.000)	2 (4.348)	
*Ultrasound hypoechoic nodules*	Negative	46 (31.507)	30 (30.000)	16 (34.783)	0.563
	Positive	100 (68.493)	70 (70.000)	30 (65.217)	
*Prostatic calculi*	Negative	66 (45.205)	45 (45.000)	21 (45.652)	0.941
	Positive	80 (54.795)	55 (55.000)	25 (54.348)	

### Univariate and multivariate logistic regression analyses

According to univariate logistic regression analysis (**Table 2**), tPSA, PSAD, F/T, BMI, LSR, RDW, and blood glucose level were significant predictors of the occurrence of PCa in the overall population (P > 0.05). PSA and PSAD are significantly correlated, and the univariate analysis indicated that PSAD was more statistically significant as a predictor than PSA. Therefore, we only included PSAD in the multivariate analysis. The significant characteristics identified from the univariate analysis above were included in multivariate logistic regression analysis (**Table 3**). The odds ratios (ORs) calculated indicated the relative risk of PCa. The results showed that PSAD (OR = 11.539, 95% CI = 4.388–33.993), LSR (OR = 0.189, 95% CI = 0.059–0.561), BMI (OR = 2.638, 95% CI = 1.067–6.871), and urine RBC level (OR = 0.136, 95% CI = 0.018–0.620) were independent predictors of PCa. In addition, ALP (OR = 6.00, 95% CI = 1.052–34.212) was also identified as a significant independent predictor (P = 0.044) in univariate logistic regression analysis, but it was not included in further analysis as there were too many missing values (n = 31), **Supplementary Tables 2, 3**.

**Table 2:** Univariate logistic regression in the differential diagnosis of prostate cancer in the whole data cohort.

Variables	OR	95%CI	P-value
*PSA;ng/mL*			
*<8.47*	1(reference)		
*≥8.47*	2.162	[1.019,4.590]	0.045
*fPSA;ng/mL*			
*<1.89*	1(reference)		
*≥1.89*	0.535	[0.243,1.179]	0.121
*F/T*			
*<0.103*	1(reference)		
*≥0.103*	0.331	[0.146,0.750]	0.008
*PV;mL*			
*<38.1*	1(reference)		
*≥38.1*	0.109	[0.049,0.243]	<0.001
*PSAD*			
*<0.24*	1(reference)		
*≥0.24*	8.267	[3.761,18.169]	<0.001
*Age;years*			
*<73*	1(reference)		
*≥73*	2.232	[0.959,5.194]	0.062
*BMI;kg/m²*			
*<23.62*	1(reference)		
*≥23.62*	2.062	[1.011,4.204]	0.046
*NLR*			
*<1.46*	1(reference)		
*≥1.46*	0.484	[0.210,1.115]	0.088
*PLR*			
*<131.01*	1(reference)		
*≥131.01*	1.371	[0.676,2.779]	0.382
*MLR*			
*<0.336*	1(reference)		
*≥0.336*	0.716	[0.350,1.467]	0.362
*GLR*			
*<13.21*	1(reference)		
*≥13.21*	0.587	[0.290,1.190]	0.14
*LSR*			
*<0.684*	1(reference)		
*≥0.684*	0.357	[0.159,0.802]	0.013
*RPR*			
*<0.00063*	1(reference)		
*≥0.00063*	0.513	[0.253,1.040]	0.064
WBC;*10⁹ */L*			
*<4.89*	1(reference)		
*≥4.89*	0.423	[0.176,1.020]	0.055
*Hb;g/dl*			
*<146.2*	1(reference)		
*≥146.2*	0.338	[0.109,1.045]	0.06
*RDW; %*			
*<0.15*	1(reference)		
*≥0.15*	0.194	[0.043,0.871]	0.032
Plt;*10⁹ */L*			
*<207*	1(reference)		
*≥207*	1.539	[0.760,3.119]	0.231
Neutrophil count;*10⁹ */L*			
*<4.14*	1(reference)		
*≥4.14*	0.626	[0.304,1.290]	0.204
Lymphocyte count;*10⁹ */L*			
*<1.5*	1(reference)		
*≥1.5*	0.617	[0.285,1.337]	0.221
Monocyte count;*10⁹ */L*			
*<0.44*	1(reference)		
*≥0.44*	0.595	[0.264,1.343]	0.212
Eosinophil count;*10⁹ */L*			
*<0.17*	1(reference)		
*≥0.17*	0.51	[0.251,1.035]	0.062
*Blood glucose;mmol/L*			
*<5*	1(reference)		
*≥5*	0.493	[0.243,1.002]	0.05
*γ-glutamyl transpeptidase;U*			
*<33.2*	1(reference)		
*≥33.2*	1.978	[0.955,4.097]	0.066
*Creatinine;μmol/L*			
*<88*	1(reference)		
*≥88*	1.496	[0.739,3.028]	0.263
*ALP;U*			
*<90*	1(reference)		
*≥90*	6	[1.052,34.212]	0.044
*Urine WBC*			
*Negative*	1(reference)		
*Positive*	0.366	[0.130,1.028]	0.056
*Urine RBC*			
*Negative*	1(reference)		
*Positive*	0.152	[0.034,0.676]	0.013
*Ultrasound hypoechoic nodules*			
*Negative*	1(reference)		
*Positive*	0.804	[0.382,1.688]	0.564
*Prostatic calculi*			
*Negative*	1(reference)		
*Positive*	0.974	[0.483,1.964]	0.941

**Table 3:** Multivariate logistic regression in the differential diagnosis of prostate cancer in the whole data cohort.

Variables	OR	95%CI	P-value
*PSAD*			
*<0.24*	1(reference)		
*≥0.24*	11.539	(4.388,33.993)	<0.001
*F/T*			
*<0.103*	1(reference)		
*≥0.103*	0.848	(0.294,2.515)	0.762
*LSR*			
*<0.684*	1(reference)		
*≥0.684*	0.189	(0.059,0.561)	0.004
*BMI;kg/m²*			
*<23.62*	1(reference)		
*≥23.62*	2.638	(1.067,6.871)	0.04
*RDW; %*			
*<0.15*	1(reference)		
*≥0.15*	0.259	(0.036,1.156)	0.111
*Blood glucose;mmol/L*			
*<5*	1(reference)		
*≥5*	0.501	(0.192,1.269)	0.148
*Urine_RBC*			
*Negative*	1(reference)		
*Positive*	0.136	(0.018,0.62)	0.022

### Performance of ML algorithms

**Figure 2:**
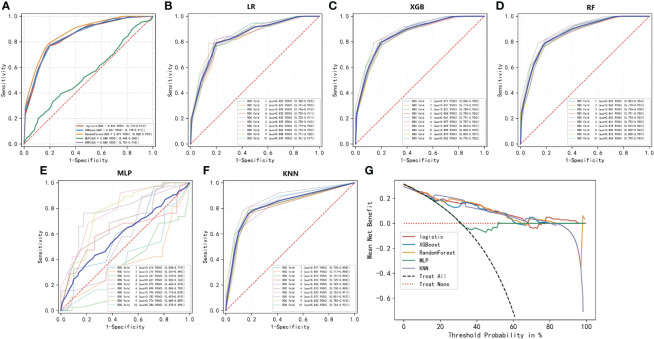
ROC and decision curve analyses of the five ML algorithms. **(A–F)** ROC curve analysis of a ten-fold cross-validation of five machine learning algorithms for predicting the risk of PCa in the training cohort. **(G)** Decision curve analysis demonstrating the net benefit associated with the use of the models for the prediction of upstaging.

**Figure 3:**
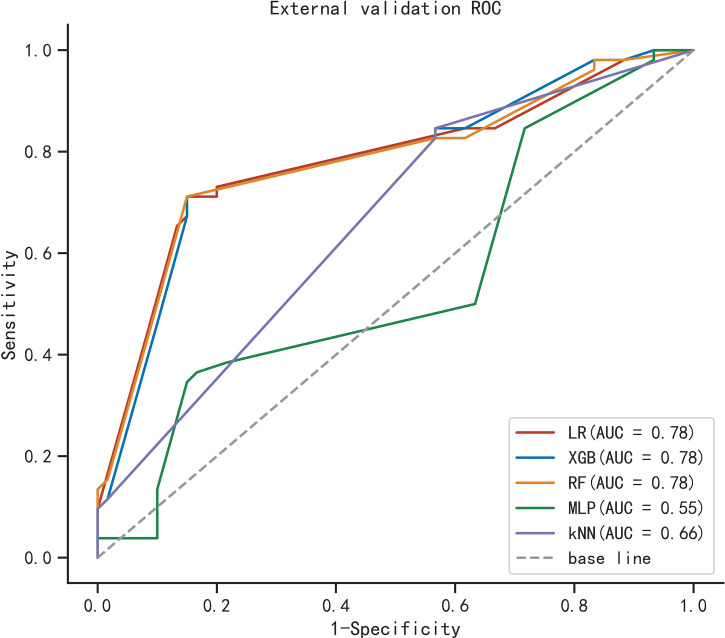
ROC curve analysis of five machine learning algorithms for predicting the risk of PCa in the external cohort.

To compare the predictive performance of the six ML algorithm models, ten-fold cross validation and decision curve analysis was applied (**Figure 2**). As shown in the figure, the RF model exhibited the best performance in the differential diagnosis of PCa, with an average AUC of 0.871 (95% CI = 0.808–0.933). The ML algorithm-based models outperformed PSA and its derivatives F/T and PSAD individually by a significant margin (AUC of PSA = 0.589, AUC of F/T = 0.599, AUC of PSAD = 0.737). Therefore, the RF model was finally regarded as the preferred prediction model. In the external validation group (**Figure 3**), RF (AUC = 0.780, 95% CI = 0.691–0.869), LR (AUC = 0.781, 95% CI = 0.692–0.871) and XGB (AUC = 0.780; 95% CI = 0.692–0.868) showed good AUC values of 0.780. Based on the findings for the training cohort and the external validation cohort together, we finally choose the RF algorithm model as the best model and used it for further analysis.

### Relative importance of the analyzed variables

**Figure 4:**
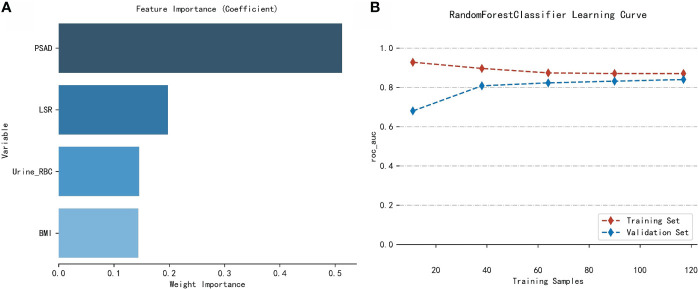
Analysis of the RF model. **(A)** Importance of the variables included in the RF model in the training cohort. **(B)** Learning curves of the RF model in the training cohort.

The importance of the included features based on the RF algorithm differed from each other, and PSAD was identified as the most important variable. They were arranged as follows in descending order of importance: PSAD, LSR, urine RBC level, and BMI (**Figure 4**).

## Discussion

One of the main topics of research on urological prostate cancer is the improvement of prediction accuracy before prostate puncture biopsy in order to reduce unnecessary patient pain without compromising on early intervention in patients with a confirmed diagnosis. The study variables included in this study were non-invasive, and data on these variables were readily available prior to biopsy. Therefore, obtaining the data for these variables did not involve any unduly demanding conditions or excessive medical overhead. Previous studies have mostly been limited to PSA and its derivatives, and there are not enough studies on the differential value of other inflammatory markers (26–28). Further, the currently used prediction nomogram based on the conventional algorithm also has room for further improvement.

The clinical application of ML algorithms may facilitate a paradigm shift in the medical field, as these algorithms are efficient, objective, and reproducible when it comes to large amounts of nonlinear data (24, 29–32). They also have the potential to improve the quality of early diagnosis, identify disease progression, and increase the likelihood of predicting patient-specific outcomes (25, 33, 34). These advantages can facilitate the sharing of information for decision-making between clinicians and patients and promote efficient planning and visualization of the use of healthcare services. In addition, the model can be actively retrained over time to continuously improve its own predictive accuracy.

The AUC value of our RF algorithm was 0.871 in the training cohort, and this value is significantly higher than the individual AUC values of PSA (AUC = 0.589), F/T PSA (AUC = 0.599), and PSAD (AUC = 0.737). The RF-based model performed well in the external validation cohort, with an AUC value of 0.78, a sensitivity of 0.712, and a specificity of 0.85. Decision curve analysis was used to validate the efficacy and potential benefits of our novel model. This ML-based model can be used as a screening tool for prostate biopsy and has the potential to avoid missed diagnosis of PCa. Four independent predictors for PCa diagnosis were identified in our analysis: PSAD, BMI, LSR, and urine RBC level. Previous studies have suggested that obese patients have a higher risk of developing prostate cancer. This is probably because periprostatic fat is biologically active and can secrete factors that promote cancer growth. However, it is unclear whether reversing obesity can mitigate the progression of prostate cancer (35–38). The presence of a visible hematuria is a common sign of prostatic disease. We believe that urine RBC level emerged as a predictive factor in this study because there was a high percentage of patients with benign lesions, and prostatic hyperplasia is associated with a lower incidence of urinary tract symptoms. Difficulty in urination can cause damage to the microvasculature of the urinary system, and this can manifest as urine occult blood. In contrast, PCa in its early clinical stage is often insidious, and most patients only seek treatment when elevated PSA is detected during routine physical examination. In previous studies, LSR has been applied in the evaluation of gestational diabetes (21), diagnosis of cirrhosis (22), and prognosis of different cancers (39, 40). The levels of alanine aminotransferase and aspartate aminotransferase may be affected by obesity (41, 42), and fluctuations in these two indicators may influence the diagnosis of PCa in a similar way that BMI influences PCa. Although ALP was not further analyzed in the current study due to missing data, ALP may still be a promising indicator for the diagnosis of prostate cancer. Further, it has been suggested that prostate cancer may exhibit overexpression of tumor-derived ALP, but this needs to be validated in further studies (43)

Our study has several limitations. First, the small sample size may affect the conclusions of the statistical analysis. Second, our study was a single-center retrospective analysis, so there is a possibility of a selection bias that may have affected the accuracy of our model. Future external validation is needed to assess the clinical application of our ML model by using data from other institutions. Finally, some meaningful indicators may not have been included in our analysis because of the absence of some hematological data, and this may have affected the efficacy of the model. Therefore, these findings need to be confirmed in future investigations on larger patient samples.

## Conclusion

We established an efficient ML model for the differential diagnosis of PCa. Our model exhibited excellent predictive accuracy and practical clinical utility, and may help guide the decision-making process of the urologist, avoid unnecessary prostate puncture biopsy, and increase the detection rate of PCa.

## Data availability statement

The original contributions presented in the study are included in the article/**Supplementary Material**. Further inquiries can be directed to the corresponding author.

## Ethics statement

This study was approved by the institutional review board of the First Affiliated Hospital of Guangxi Medical University. Written informed consent was obtained from all patients for the storage of their information for the purpose of research. All the research procedures were conducted in accordance with the Declaration of Helsinki.

## Author contributions

XD, TL, LM and FW: contributed to the conception and design. XD, JJ, XH, BM and SP: collected, analyzed the data, drew the figures and tables, and wrote the draft. XD and JC contributed to manuscript writing and revision. All authors approved the final manuscript.

## Funding

This study was sponsored by Guangxi Science and Technology Base and Talent Project (Grant No. Guike AD20238090) and Guangxi Clinical Research Center for Urology and Nephrology (Grant No. Guike AD20297081).

## Conflict of interest

The authors declare that the research was conducted in the absence of any commercial or financial relationships that could be construed as a potential conflict of interest.

## Publisher’s note

All claims expressed in this article are solely those of the authors and do not necessarily represent those of their affiliated organizations, or those of the publisher, the editors and the reviewers. Any product that may be evaluated in this article, or claim that may be made by its manufacturer, is not guaranteed or endorsed by the publisher.

## Supplementary material

Supplementary Table 1 Clinical characteristics of patients in the external validation cohort.

Supplementary Table 2 Raw data from the First Affiliated Hospital of Guangxi Medical University, Guangxi, China.

Supplementary Table 3 Validation database from Changhai Hospital, Naval Medical University, Shanghai, China.
